# A Retrospective Analysis of Transfusion Management for Obstetric Hemorrhage in a Japanese Obstetric Center

**DOI:** 10.5402/2012/854064

**Published:** 2012-02-06

**Authors:** Shigetaka Matsunaga, Hiroyuki Seki, Yoshihisa Ono, Hideyoshi Matsumura, Yoshihiko Murayama, Yasushi Takai, Masahiro Saito, Satoru Takeda, Hiroo Maeda

**Affiliations:** ^1^Center for Maternal, Fetal and Neonatal Medicine, Saitama Medical Center, Saitama Medical University, 1981 Kamoda, Kawagoe, Saitama 350-8550, Japan; ^2^Department of Transfusion Medicine and Cell Therapy, Saitama Medical Center, Saitama Medical University, Kawagoe, Saitama 350-8550, Japan

## Abstract

*Background.* Since cryoprecipitate, fibrinogen concentrate, or recombinant activated factor VII is not approved by public medical insurance in Japan, we retrospectively assessed blood product usage in patients with obstetric hemorrhage at our tertiary obstetric center. *Material and Methods.* 220 patients with obstetric hemorrhagic disorders who underwent blood product transfusion in our institution during a 5-year period were reviewed for the types and volumes of blood products transfused. *Results.* There was a significant positive correlation (*P*< 0.001) between the volume of RCC (red blood cell concentrate) transfused and that of FFP (fresh frozen plasma), irrespective of underlying obstetric disorders. The median of FFP to RCC ratio in each patient was 1.3–1.4, when 6 or more units of RCC were transfused. *Conclusions.* In transfusion for massive obstetric hemorrhage in terms of appropriate supplementation of coagulation factors, the transfusion of RCC : FFP = 1 : 1.3–1.4 may be desirable.

## 1. Introduction

In the world, obstetric hemorrhage is the most common cause of maternal death, causing 24% of, or an estimated 127,000, maternal deaths annually [1]. It has also been reported that massive (2,000 mL or more) and life-threatening obstetric hemorrhage occurs in 3–5% [2] and 0.1% [3] of deliveries, respectively, and blood product transfusion is required in 0.3–1% [2, 4]. Also in Japan, obstetric hemorrhage account causes 25% of maternal deaths [5] and massive obstetric hemorrhage (MOH) occurs in 1.1% of deliveries [6].

 In the terminal stage of pregnancy, where the coagulation system is enhanced and the fibrinolysis system is inhibited [7, 8], MOH may be apt to induce consumptive loss of coagulation factors, which causes further hemorrhage, forming a vicious circle such as disseminated intravascular coagulation (DIC) [9]. Accurate evaluation of blood loss is important to determine whether transfusion should be performed, but it is difficult in obstetric hemorrhage [10–12]. In addition, high-level capacity of pregnant women to tolerate obstetric hemorrhage [13, 14] masks changes in their vital signs, resulting in a delay in the detection and treatment of hypovolemia, which causes further hemorrhage and hemorrhagic shock. Therefore, the comprehensive evaluation of not only blood loss but also the cause of hemorrhage, a patient's medical condition, age, vital signs, and blood biochemical data is required to determine whether transfusion is necessary [15].

 Understanding the above-mentioned specificity of obstetric hemorrhage is also required for appropriate blood product support, which can effectively improve its pathophysiological condition, reduce the risk of DIC, and avoid the aggravation of hemorrhagic shock [16]. However, in Japan, “Principles for blood transfusion therapy” and “Principles of the use of blood products” [17] proposed by the Japanese Ministry of Health, Labour and Welfare give little consideration to the pathophysiological mechanism or no standard for the appropriate dosage of blood product transfusion specific to obstetric hemorrhage. Therefore, we retrospectively reviewed blood product administration in patients with obstetric hemorrhage at our tertiary obstetric center for a 5-year period and discuss appropriate dosage of blood product transfusion for obstetric hemorrhage.

## 2. Material and Methods

### 2.1. Subjects

Between January 1, 2004, and December 31, 2008, 243 obstetric patients underwent blood product transfusion in our tertiary perinatal institution, Saitama Medical Center/Saitama Medical University, which is only general medical institution for tertiary perinatal care in Saitama prefecture with population of 7.2 million. Their data were manually abstracted by our research staffs from our medical records, anonymized in an unlinkable fashion prior to our investigation, which exempted us from institutional review board approval according to “Ethical principles for etiological studies” [18] proposed by the Japanese Ministry of Health, Labour and Welfare.

 Blood products involved in this study are red cells concentrates (RCC), fresh-frozen plasma (FFP), and platelet concentrates (PC). Two units of RCC (approximately 140 mL/unit), 3 units of FFP (approximately 80 mL/unit), and 2 units of PC (approximately 20 mL/unit) are derived from 400 mL of whole blood, respectively, in this study.

### 2.2. Our Management Principles for Blood Product Transfusion (Table 1)

Since blood loss in vaginal delivery or Caesarean section is difficult to evaluate accurately [11, 12] and hemoglobin (Hb) concentration necessary to maintain appropriate hemodynamics and oxygen supply is ≥7 g/dL [19, 20], Hb concentration <7 g/dL was determined to be an indication for blood product transfusion in principle. In addition to this principle, the patient's age, medical condition, state of hemorrhage, and blood test data were taken into consideration [8, 15]. Since the transfusion for patients with an Hb concentration ≥7 g/dL and stable vital signs may lead to excessive transfusion, RCC transfusion was performed with a goal Hb concentration of 7-8 g/dL [21]. FFP was concomitantly transfused until the coagulation function normalizes [9, 22]. We did not have any rule in advance to define the proportion of FFP to RCC in the present study.

 Cryoprecipitate, fibrinogen concentrate, or recombinant activated factor VII was not administered in general, since they are not approved or paid by public medical insurance in Japan.

### 2.3. Evaluated Items

The following items were retrospectively evaluated: underlying disorders which required blood product transfusion, types of blood product and their transfused volume, and data of hemoglobin (Hb) concentration, percent prothrombin activity (%PT; normal range: 84–117% in our institution), activated partial thromboplastin time (aPTT; 25–36 sec), and fibrinogen concentration (150–400 mg/dL) within 30 minutes before blood transfusion. Blood test data were excluded for further statistical analyses when they were obtained after blood product was transfused.

Prothrombin activity was assayed by STA-R Evolution (Roche Diagnostics), a fully automated coagulation analyzer. Clotting times were converted to percent normal plasma prothrombin activity from a log-log standard curve prepared with dilutions of control pooled plasma [23].

### 2.4. Statistical Analyses

The presence or absence of correlations was analyzed by JMP (SAS Institute) software employing Spearman's rank correlation coefficient, and equality of parameters among groups was analyzed employing Kruskal-Wallis one-way analysis of variance by ranks because each parameter did not show normal distribution. In each test, *P* < 0.05 was regarded as significant.

## 3. Results

### 3.1. Obstetric Patients Who Underwent Blood Product Transfusion (Table 2)

We have experienced 243 obstetric patients who underwent blood product transfusion, consisting of 164 (67%) delivered by Cesarean section and 79 (33%) delivered vaginally. Eighty-two (34%) women were transported to our institution in their puerperium for our specialized management of obstetric hemorrhage. For 8 patients who had blood transfusion prior to or during transfer, we included the data on transfusion using their medical record of transfer source institution.

 We had one patient who died with amniotic fluid embolism resulting in severe coagulopathy and multiple organ failure and one vegetative patient with severe HELLP syndrome resulting in hypoxic encephalopathy.

 Two hundred and twenty (91%) patients underwent blood transfusion for obstetric hemorrhage, while 17 (7%) patients for hematological disorders during pregnancy such as idiopathic thrombocytopenic purpura (ITP), myelodysplastic syndrome (MDS), and leukemia. Four other patients underwent blood transfusion for major disorders during pregnancy such as cerebral infarction, gastric cancer, colon cancer, and head injury. One pregnant woman with cardiac dysfunction and one with no routine checkup but severe anemia had blood transfusion in their emergency Cesarean delivery.

### 3.2. Obstetric Indications of Blood Transfusion (Table 3)

Table 3 shows the 220 cases with obstetric hemorrhage that required blood product transfusion. Most of the cases underwent blood transfusion in their peripartum, except for a few cases who had massive hemorrhage several days after their delivery.

 Some of the uterine atony cases resulted from retained placental tissue or overdistension due to multiple pregnancy. Genital tract trauma was noted in the vaginal wall, cervix, and/or uterine body (uterine rupture was detected in 6 cases). Thirteen cases of placenta previa were complicated by placenta acreta, increta, or percreta.

### 3.3. Blood Product Transfusion for Obstetric Indications

Table 4 shows the number of cases and median volumes for each blood product transfused for the 220 cases with obstetric hemorrhage shown in Table 3. No cryoprecipitate, fibrinogen concentrate, or recombinant factor VII was administered. Autologous whole blood was transfused for 24 patients with placenta previa with or without placenta acreta, increta, or percreta. These patients who had autologous blood transfusion were excluded for the analysis.

**Figure 1:**
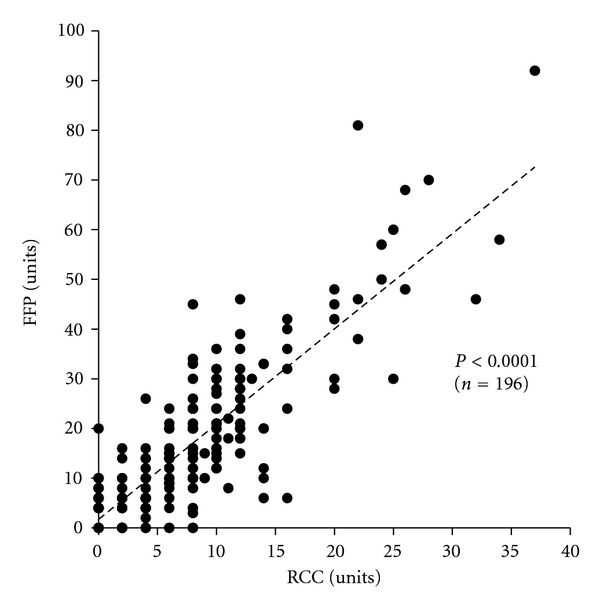
Correlation between the volume of RCC and FFP in obstetric hemorrhage patients with allogenic transfusion (*n* = 196). A significant positive correlation was observed between the volume of RCC and that of FFP, as also shown in Table 5. Patients who underwent autologous transfusion were excluded. An outlier with amniotic fluid embolism who had 50 units of RCC and 116 units of FFP was also excluded.

 In 196 patients with obstetric hemorrhage who underwent only allogenic transfusion, there was a significant positive correlation (*P* < 0.001) between the volume of RCC transfused and that of FFP (Figure 1). This significant positive correlation was shown irrespective of underlying obstetric disorders (Table 5).

 A ratio of total transfused units of FFP to RCC was 2.1 (3,550/1,665) in the 196 patients with allogenic transfusion alone. The median of FFP/RCC ratio for each patient was 2.0 in total (Table 5), and 2.1 and 2.0 in subgroups with moderate (6–9 units) and massive (10 units or more) RCC transfusion, respectively (Table 6). The median of FFP/RCC ratio was not significantly different between underlying obstetric disorders, but 2.0 or more except for uterine atony (Table 5).

### 3.4. Comparison of Blood Test Data among Underlying Obstetric Disorders

Next, we compared Hb concentration, percent prothrombin activity (%PT), aPTT, and fibrinogen concentration immediately before blood transfusion among underlying obstetric disorders which required blood product transfusion. From the 220 obstetric disorder cases, 8 patients who had our blood tests only after transfusion were excluded for the analysis. One patient with amniotic fluid embolism was also excluded.

**Figure 2:**
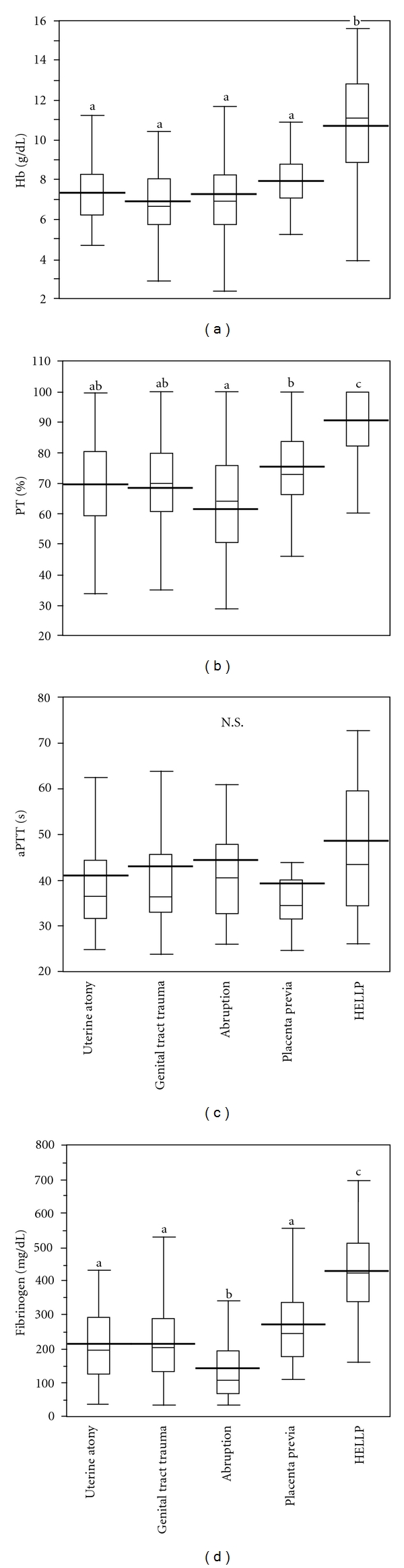
Hemoglobin (Hb) concentration, percent prothrombin activity (%PT), activated partial thromboplastin time (aPTT), and fibrinogen concentration immediately before blood transfusion in each obstetric hemorrhagic disorder (*n* = 211). Box plot graphs represent the median value as well as the upper and lower quartiles. The line across the middle of the box identifies the median sample value. The whiskers extend from the ends of the box to the outermost data point. The thick lines denote the mean values. Different superscript letters (a, b, c) denote significant difference at *P* < 0.05. Patients were excluded as mentioned in the text.

 As shown in Figure 2, aPTT was not significantly different among obstetric disorders. Hb concentration was significantly higher in HELLP syndrome compared with other disorders. %PT in placental abruption was not significantly different from those in uterine atony/inversion or genital tract trauma, but significantly lower compared with placenta previa and HELLP syndrome. Fibrinogen concentration in placental abruption was significantly lower, and that in HELLP syndrome was significantly higher compared to other disorders.

### 3.5. Correlation between Data of Blood Tests and the Volumes of Blood Products Transfused

To clarify the characteristics of hemorrhage in total or each underlying obstetric disorders, we evaluated the possible correlation between data from blood tests and the volumes of blood products transfused. As mentioned previously, 32 patients who had our blood tests only after transfusion or autologous blood transfusion were excluded for the analysis. One maternal death with amniotic fluid embolism was also excluded since severe coagulopathy resulting in MOF was not controlled by massive blood product transfusion.

#### 3.5.1. Correlation between the Volume of RCC Transfusion and Hb Concentration or %PT

**Figure 3:**
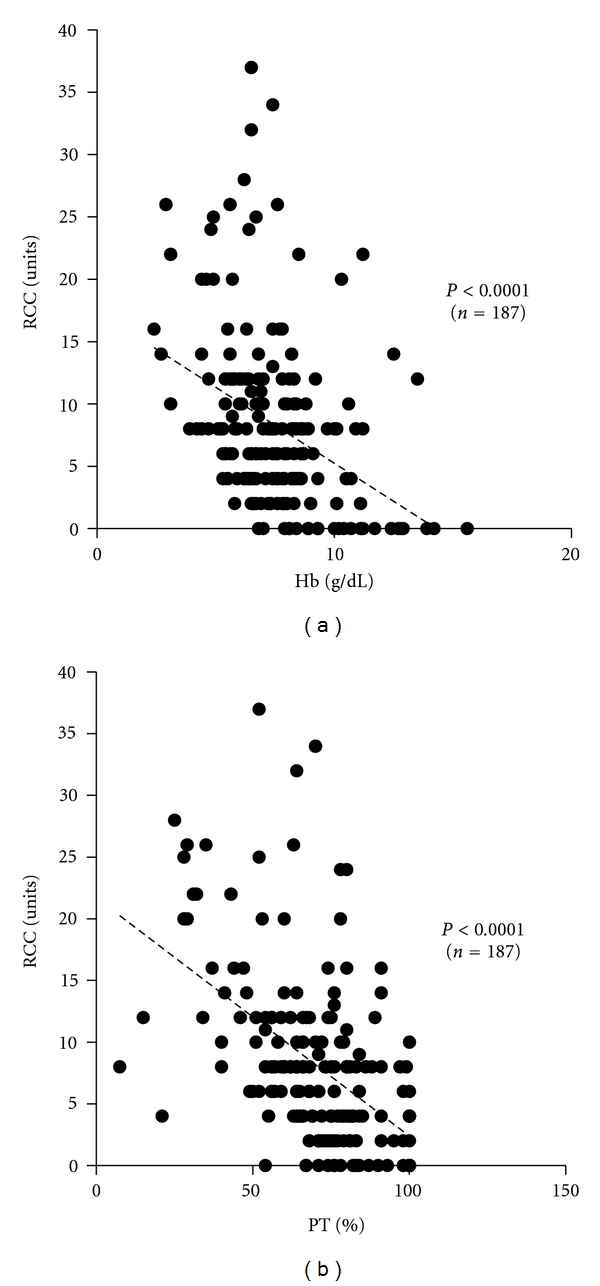
Correlation between the volume of RCC transfused and Hb concentration or %PT in obstetric hemorrhage patients (*n* = 187). A significant negative correlation was observed between the volume of RCC transfused and Hb (a), or RCC and %PT (b). Patients were excluded as mentioned in the text.

In the 187 obstetric disorder patients, there was a significant negative correlation (*P* < 0.001) between the volume of RCC transfused and Hb concentration immediately before transfusion (Figure 3). This significant negative correlation was shown irrespective of underlying obstetric disorders except for placenta previa (data not shown).

 Similarly, there was a significant negative correlation (*P* < 0.001) between the volume of RCC transfused and %PT (Figure 3). This significant negative correlation was shown irrespective of underlying obstetric disorders except for placenta previa and HELLP syndrome (data not shown).

#### 3.5.2. Correlation between the Volume of FFP Transfusion and %PT or Fibrinogen Concentration

**Figure 4:**
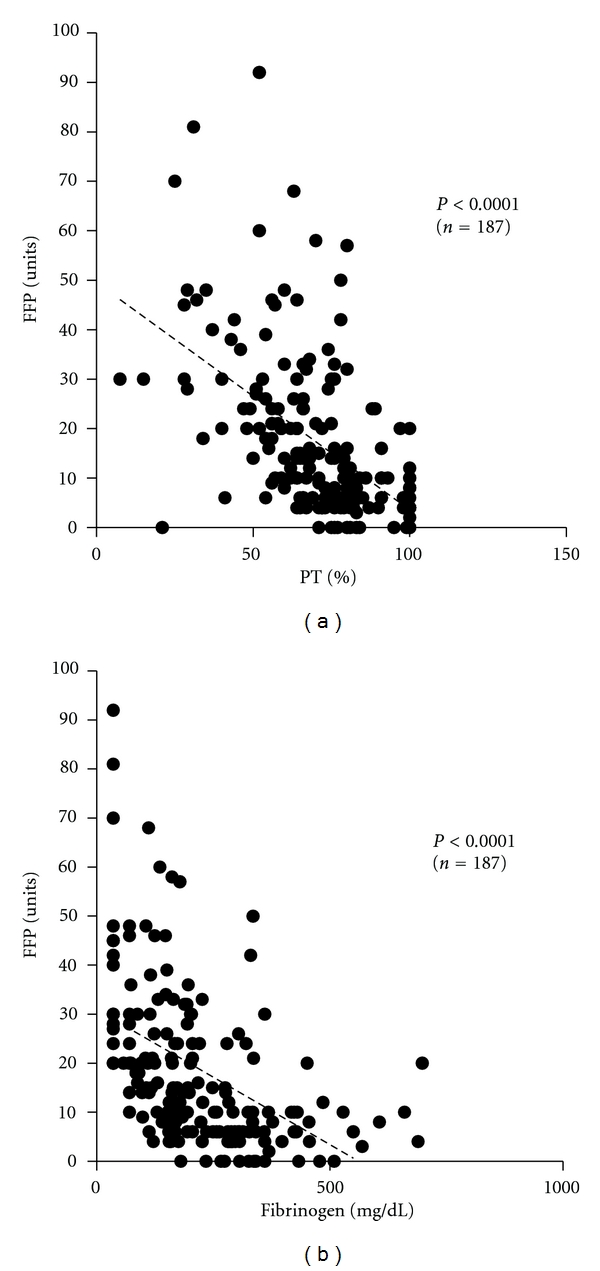
Correlation between the volume of FFP transfused and %PT or fibrinogen concentration in obstetric hemorrhage patients (*n* = 187). A significant negative correlation was observed between the volume of FFP transfused and %PT (a), or FFP and fibrinogen (b). Patients were excluded as mentioned in the text.

Also in FFP transfusion, there was a significant negative correlation (*P* < 0.001) between the volume of FFP transfused and %PT immediately before transfusion (Figure 4). This significant negative correlation was shown irrespective of underlying obstetric disorders except for placenta previa (data not shown).

 Similarly, there was a significant negative correlation (*P* < 0.001) between the volume of FFP transfused and fibrinogen concentration (Figure 4). This significant negative correlation was shown irrespective of underlying obstetric disorders except for placenta previa and HELLP syndrome (data not shown).

## 4. Discussion

From 2004 to 2008, the incidence of blood product transfusion for obstetric patients in our tertiary perinatal institution was 4.6% (243/5,311), which was much higher than previously reported incidences, 0.3–1% [2–4]. In these patients who had blood product transfusion, the percentage of patients with 6 or more units of RCC was 57% (138/243), which was also higher than previously reported, 32–42% [2, 4, 24]. This may be because our institution is only general medical institution for tertiary perinatal care in Saitama prefecture with population of 7.2 million and has accepted obstetric patients with life-threatening conditions.

 Of the 220 patients with obstetric hemorrhage who underwent blood product transfusion (Table 3), FFP was transfused for 92.3% (203/220) (Table 4), suggesting importance of coagulation factors in blood transfusion for obstetric hemorrhage. In the patients with obstetric hemorrhage who underwent only allogenic transfusion, there was a significant positive correlation between the volume of RCC transfused and that of FFP (Figure 1) irrespective of underlying obstetric disorders (Table 5). A ratio of total transfused units of FFP to RCC and the median of FFP/RCC ratio in each patient was 2.1 and 2.0 (Table 5), respectively. Moreover, the median FFP/RCC ratio was 2.0 and 2.1 in subgroups with moderate and massive RCC transfusion, respectively (Table 6). Since two units of RCC and 3 units of FFP are derived from 400 mL of whole blood, respectively, in this study, the RCC : FFP ratio of 1 : 2.0–2.1 in units is equivalent to 1 : 1.3–1.4 when these volumes are converted to whole blood. This RCC : FFP ratio of 1 : 1.3–1.4 is almost consistent with the report of Borgman et al. who recommended the transfusion of RCC and plasma at a ratio of 1 : 1.4 for massive hemorrhage [25].

 To evaluate possible differences among obstetric hemorrhagic disorders in their pathophysiological condition which may affect the volumes of RCC and FFP transfused, we compared Hb concentration, %PT, aPTT, and fibrinogen concentration immediately before blood transfusion. Placental abruption causes dissection of blood at the decidual-placental interface, resulting in entry of placental tissue factor into the circulation to promote thrombin generation which eventually leads to disseminated intravascular coagulation (DIC) [26]. As shown in Figure 2, fibrinogen concentration is significantly lower in placental abruption compared with other obstetric disorders, suggesting severer coagulopathy than other obstetric disorders.

 As mentioned previously, accurate evaluation of blood loss is difficult in obstetric hemorrhage [10–12], so comprehensive evaluation of not only blood loss but also the cause of hemorrhage and blood test data is required to determine the timing and volume of blood transfusion [15]. Moreover, our present data lack some other important variables prior to blood transfusion, such as rates and volumes of IV fluid resuscitation and use of surgical or pharmacologic interventions. Nonetheless, as shown in Figure 3, there was a significant negative correlation between the volume of RCC transfused and Hb concentration immediately before transfusion. This significant negative correlation was shown in all obstetric disorders except for placenta previa. This may be because for some patients with placenta previa the interval between onset of hemorrhage and blood test was so short that data did not reflect blood loss, and the transfusion volume was determined by intraoperative blood loss count.

 Similarly, there was a significant negative correlation between the volume of FFP transfused and %PT or blood fibrinogen concentration (Figure 4), showing that patients with severer coagulation factor depletion required a larger volume of FFP for its supplementation. Interestingly, there was a significant negative correlation also between the volume of RCC transfused and %PT as data of coagulation system tests (Figure 3), suggesting that coagulation dysfunction due to coagulation factor depletion may result in even more blood loss and increase in transfusion volume of not only FFP but also RCC. These results are consistent with studies showing an increase in blood loss due to secondary atonic bleeding unless coagulation factors are rapidly supplemented [27], further dilution of coagulation factors and blood loss resulting from RCC and extracellular fluid supplementation alone without coagulation factors to pregnant and puerperal women with MOH [28], an increase in blood loss along with a decrease in coagulation factors, particularly with blood fibrinogen level less than 200 mg/dL [29], and the indispensability of coagulation factor supplementation when the blood fibrinogen level is ≤100 mg/dL [30]. Appropriate supplementation of coagulation factors normalizes the coagulation function in the early stage and reduces not only blood loss but also the volume of blood product transfusion [22, 27, 28, 31].

 According to Figures 3 and 4, it is also noted that considerable number of patients with Hb, %PT, and fibrinogen values in the normal range were transfused with RCC and/or FFP. This may be because above-mentioned comprehensive evaluation of blood loss, cause of hemorrhage, vital signs, underlying disorders, and so forth has led to the clinical decision for blood product transfusion before the deterioration of their blood test values. On the other hand, Table 6 shows that 24 (12%) patients received FFP without RCC. In these patients consisting of 5 with placental abruptions, 7 with uterine atonies, 1 with genital tract trauma, and 11 with HELLP syndromes, RCC transfusion was not required since coagulation factors were promptly supplemented with FFP and their Hb levels could be maintained above 7 g/dL. Although some retrospective analyses reported that a percentage of patients were inappropriately transfused [24, 32], we believe that prompt decision making is inevitable to avoid secondary atonic bleeding and DIC especially in life-threatening obstetric hemorrhage.

 In conclusion, for massive obstetric hemorrhage where appropriate supplementation of coagulation factors is essential, the transfusion of RCC : FFP = 1 : 1.3–1.4 in terms of whole blood is desirable according to our retrospective analysis as well as previous report [25].

## Figures and Tables

**Table 1:** Our transfusion management principles.

(1) While evaluating the bleeding state, consumed coagulation factors are rapidly supplemented until the coagulation function normalizes [9, 22]	
(2) With the above as a goal, 4–6 units of FFP are transfused at a time, and the coagulation function is evaluated after each transfusion	
(3) Stabilization of the vital signs	
(4) RCC transfusion is performed to achieve an Hb level of 7-8 g/dL as a goal	
(5) PC transfusion is performed to achieve a platelet count above 50,000/mm^3^ as a goal	
(6) Cryoprecipitate, as well as specific coagulation factor preparations, is not administered in general	

**Table 2:** Demographics of obstetric patients with blood transfusion (*n* = 243).

Age in years, mean ± SD	32.1 ± 4.7
Gestational age in weeks, mean ± SD	35.3 ± 4.9
Primipara, *n* (%)	95 (39.1)
Multiple pregnancy, *n* (%)	14 (5.8)
Cesarean delivery, *n* (%)	164 (67.5)
Assisted vaginal delivery, *n* (%)	26 (10.7)

**Table 3:** Obstetric hemorrhagic disorders with blood transfusion (*n* = 220).

	*n* (%)
Uterine atony	57 (25.9)
Genital tract trauma including uterine rupture/injury	51 (23.2)
Placental abruption	48 (21.8)
Placenta previa without acreta/increta/percreta	30 (13.6)
Placenta previa with acreta/increta/percreta	13 (5.9)
Uterine inversion	5 (2.3)
HELLP syndrome	15 (6.8)
Amniotic fluid embolism	1 (0.5)

**Table 4:** Blood products transfused for obstetric hemorrhage (*n* = 220).

Blood product	*n* (%)	Median units (range)
Red cell concentrate	188 (85.5)	8 (2–50)
Fresh frozen plasma	203 (92.3)	14 (2–116)
Platelet concentrate	62 (28.2)	20 (10–80)
Autologous whole blood	24 (10.9)	3 (1–8)

**Table 5:** Significant positive correlation between RCC and FFP and an FFP/RCC ratio in each obstetric hemorrhagic disorder.

	*n*	Spearman's rank correlation coefficient (*ρ*)	*P*	FFP/RCC*
Uterine atony/inversion	62	0.7843	<0.0001	1.5 (1.2–2.5)
Genital tract trauma	51	0.7841	<0.0001	2.3 (1.5–2.9)
Placental abruption	48	0.7818	<0.0001	2.3 (1.5–3.0)
Placenta previa	19	0.7765	<0.0001	2.0 (1.1–2.1)
HELLP syndrome	15	0.5290	0.0426	2.0 (1.9–2.8)

Total	196	0.7769	<0.0001	2.0 (1.4–2.5)

*Data shown are median (interquartile range).

**Table 6:** FFP transfusion volume and an FFP/RCC ratio in obstetric hemorrhage patients with minimal, moderate, and massive RCC transfusion.

RCC transfusion	*n*	FFP (units)*	FFP/RCC*
None	24	5 (4–8)	—
Minimal (2–4 units)	49	6 (4–8)	1.5 (1.0–3.0)
Moderate (6–9 units)	53	14 (10–20)	2.0 (1.3–2.7)
Massive (10- units)	70	30 (20–42)	2.1 (1.5–2.5)

*Data shown are median (interquartile range).
